# 

**Published:** 1897-01

**Authors:** 


THE meeting of the Southern Section of the American Laryngological, Rhinological, and Otological Society will be held in New Orleans, March 3d and 4th, 1897. This date has been selected, as it will permit visiting members to see New Orleans during the Carnival season, and will enable them to secure half-rate railroad transportation.

A meeting of a laryngological, rhinological, and otological society in the South is a distinctly new enterprise, and should be encouraged. With the active co-operation of the physicians interested in this work, the Carnival meeting of the Southern Section of the American Laryngological, Rhinological, and Otological Society will be an assured success.

Candidates for membership should send their names, properly indorsed, to Dr. Robert C. Myles, Secretary, 46 West 38th St., New York, or to Dr. W. Scheppegrell, Medical Building, New Orleans, so that they may be acted upon by the Council of the Society.

